# Orbital neuroblastoma metastasis: A case report and literature review

**DOI:** 10.1097/MD.0000000000017038

**Published:** 2019-09-06

**Authors:** Wan-Ju Yang, Yuan-Yuan Zhou, Fang Zhao, Zhong-Ming Mei, Shuang Li, Yi Xiang

**Affiliations:** Department of Ophthalmology, The Central Hospital of Wuhan, Tongji Medical College of Huazhong University of Science and Technology, Wuhan, Hubei, China.

**Keywords:** adrenal tumor, case report, neuroblastoma, orbital metastasis

## Abstract

**Rationale:**

Neuroblastoma is one of the most common tumors found in children, and mostly arises in the adrenal gland and paravertebral regions. Orbital neuroblastoma metastasis is relatively rare, and is associated with poor prognosis. Since the symptoms and signs of orbital neuroblastoma are not specific, its diagnosis remains challenging.

**Patient concerns:**

A 3-year-old girl presented with periorbital ecchymoses (raccoon eyes) and proptosis for 40 days.

**Diagnoses:**

Abdominal magnetic resonance imaging (MRI) and sonography analysis revealed a large mass in the left adrenal gland (primary tumor). The computed tomography and MRI further revealed multiple soft tissue masses in the skull and both orbits with erosion of the adjacent bones (the metastasis). The histological analysis of the tumor removed from the right orbit confirmed the diagnosis of neuroblastoma.

**Interventions:**

The mass on the right face was surgically removed.

**Outcomes:**

The patient exhibited no deteriorative signs at the 6-month follow-up.

**Lessons:**

Clinical manifestations, such as periorbital ecchymoses and proptosis, in combination with radiological analysis and histological findings, are important for the diagnosis of orbital neuroblastoma metastasis.

## 1 Introduction

Neuroblastomas are the most frequently identified extracranial tumors that affect children under the age of 4 years old, with an incidence of approximately 1 to 3 in 100,000 cases.^[1,2]^ These account for approximately 6% to 10% of all pediatric tumors and 15% of tumor-related deaths in children.^[3]^ Neuroblastomas are neuroendocrine tumors that can originate anywhere in the sympathetic nervous system, although these are most commonly found in one of the adrenal glands. The clinical symptoms and signs of neuroblastomas are commonly atypical, depending on the location of the primary tumor and metastases. Although metastasis to the orbits has been well-documented for neuroblastomas presenting with various symptoms, such as periorbital ecchymoses (raccoon eyes) and proptosis,^[4]^ neuroblastoma cases with initial symptoms of orbital involvement are rare, accounting for approximately 8% of all neuroblastomas.^[5]^ In the present study, we report a specific case of a 3-year-old girl with neuroblastoma, who presented with initial signs of periorbital ecchymoses (raccoon eyes) and proptosis. In addition, the clinical, radiological, and histopathological studies of orbital neuroblastoma metastasis reported in the literature were summarized.

## 2 Case presentation

The present study was approved by the Ethics Committee of the Central Hospital of Wuhan, Tongji Medical College of Huazhong University of Science and Technology. All procedures performed in studies that involved human participants were in accordance with the ethical standards of the institutional and national research committee, and the 1964 Helsinki declaration and its later amendments, or comparable ethical standards. A written informed consent was obtained from the patient's legal guardian.

**Figure 1:**
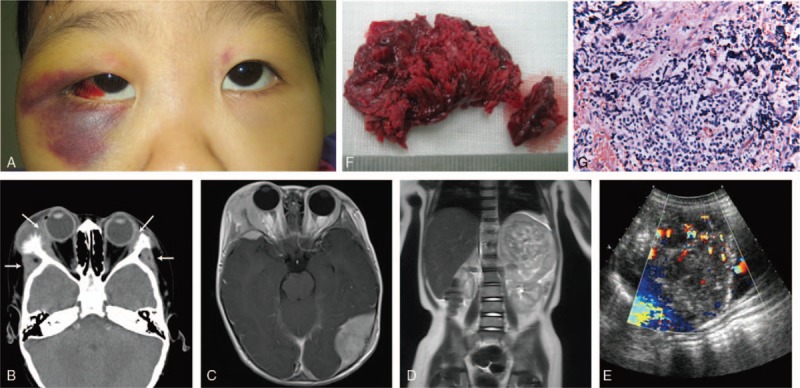
The lesion, radiological findings, and histological results of the 3-year-old girl with orbital neuroblastoma. (A) A photograph of the lesion showing the subconjunctival hemorrhages, periorbital ecchymoses (raccoon eyes), and proptosis. (B) An axial CT scan showing the bilateral proptosis and subperiosteal masses intraorbitally (narrow arrows) in the sphenoidal walls. The tumor also extended to the temporal foss (arrows). (C) The craniocerebral MRI reveals multiple hypointense soft tissue masses in the head-face area of the T1-weighted images. (D) The abdominal MRI shows a large mass lesion in the left adrenal gland protruding into the upper-middle abdomen. (E) The longitudinal abdominal sonograph shows a heterogeneous, hypoechoic mass with hyperechoic areas, and increased vascularity in the left adrenal gland. (F) The tumor removed from the right face was lobulated and hemorrhagic. (G) The H&E staining revealed uniform small, round cells with a scanty cytoplasm and darkly stained nuclei (400× magnification). CT = computed tomography, H&E staining = Hematoxylin-eosin staining, MRI = magnetic resonance imaging.

A 3-year-old girl was admitted to our hospital presenting with periorbital ecchymoses and proptosis for 40 days. On examination, multiple hard masses were palpated on both the face and head. Bilateral subconjunctival hemorrhages, periorbital ecchymoses (raccoon eyes), and proptosis were identified (Fig. 1A). Fundus fluorescein angiography (FFA) and macular optical coherence tomography (OCT) revealed no abnormality on the fundus. The systemic evaluations, which included routine blood and urine tests, hepatic and renal function examinations, and the serum glucose test, revealed unremarkable findings, with the exception of anemia. The electrocardiogram and chest roentgenogram results were also unremarkable. However, an abdominal mass was palpated by a pediatrician.

The computed tomography (CT) scan highlighted multiple soft tissue masses in the skull and both orbits, with evident erosion into the adjacent bones (Fig. 1B). The magnetic resonance imaging (MRI) also revealed multiple masses in the head-face area, and a large mass in the left suprarenal gland (Fig. 1C and D). The abdominal sonography examination revealed a heterogeneous hypoechoic mass in the left adrenal gland (Fig. 1E).

The mass on the right face was surgically removed. The tumor was cardinal red, with an irregular surface (Fig. 1F). The hematoxylin-eosin (H&E) staining revealed small uniform round cells with a scanty cytoplasm and darkly stained nuclei. Furthermore, nodules and Homer-Wright pseudorosettes were occasionally found, with no ganglionic differentiation (Fig. 1G). The tumor was immunopositive for synaptophysin and neuron-specific enolase, but immunonegative for S-100 and leukocyte common antigens. According to the clinical, radiological, and histopathological findings, the patient was diagnosed with stage IV neuroblastoma. The patient was transferred to the Department of Pediatric Oncology for the treatment of the tumor. However, the patient refused the recommended chemotherapy, which included cisplatin, doxorubicin, etoposide, and ifosfamide. Hence, the patient was discharged without any therapeutic regimen. The patient presented no deteriorative signs at the 6-month follow-up.

## 3 Discussion and conclusion

Orbital tumors in children can rapidly develop and lead to vision loss, and are associated with significant risk of mortality and morbidity.^[6]^ Neuroblastoma is an undifferentiated malignant tumor that commonly occurs in children, which can rapidly metastasize and widely spread, resulting in a variety of clinical manifestations, such as proptosis, periorbital ecchymosis, abdominal pain, anemia, pancytopenia, bone pain, and paralysis.^[7]^ In the present study, we present a case of a 3-year-old girl with stage IV neuroblastoma, who presented with periorbital ecchymoses and proptosis as initial symptoms. The diagnosis of neuroblastoma was confirmed by radiological analysis of the orbital tumors and an abdominal mass in the adrenal gland, as well as the characteristic histological findings of neuroblastoma. Since clinical manifestations and imaging features are not specific or defined for neuroblastoma, it remains difficult to differentiate neuroblastoma with orbital involvement from other metastatic orbital tumors, such as malignant lymphoma, rhabdomyosarcoma, granulocytic sarcoma, and Wilms tumors. Relevant literature was reviewed, and 10 cases of neuroblastoma with orbital involvement were found by searching PUBMED for published articles between 2000 and 2017 (Table 1). The clinical manifestations, radiological findings, and histological features of these cases, as well as the present case, were summarized (Table 1), which are integral to the clinical diagnosis and differential diagnosis of neuroblastoma with orbital involvement.

**Table 1:**
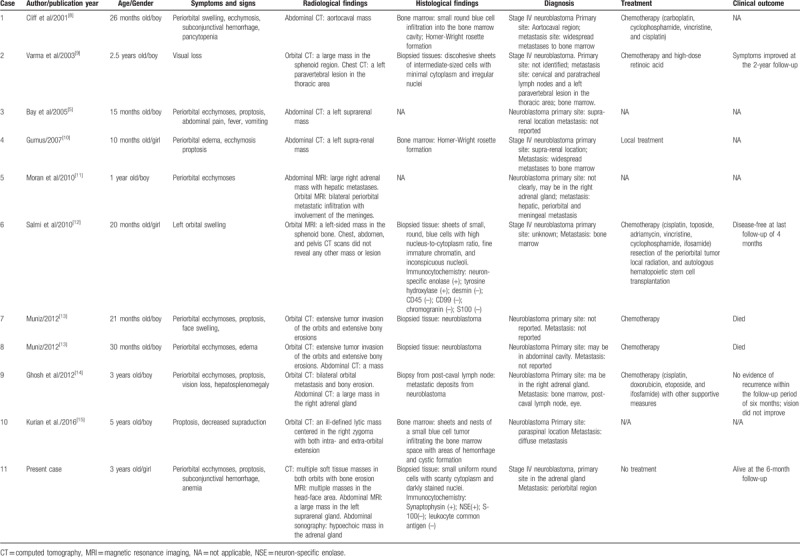
Summary of cases of orbital neuroblastoma metastases.

Periorbital ecchymoses and proptosis have been reported to be classic signs of pediatric neuroblastoma.^[4,8]^ Consistent with these symptoms, it was found that the most common clinical manifestation of neuroblastoma with orbital involvement reported in the literature was periorbital ecchymoses (8/11, 72.7%), followed by proptosis (6/11, 54.5%; Table 1). The emergence of periorbital ecchymoses is probably due to tumor obstruction of the blood vessels in and around the orbits. Less common ophthalmic manifestations include periorbital swelling/edema (5/11, 45.4%), subconjunctival hemorrhage (2/11, 18.2%), vision loss (2/11, 18.2%), and decreased ocular mobility (1/11, 9.1%). Other uncommon clinical associations include pancytopenia, anemia, abdominal pain, fever, vomiting, and hepatosplenomegaly, which may be associated with the metastasis of the tumor to the bone or other organs.

Since orbital neuroblastoma metastasis presents with non-specific clinical manifestations, it is often misdiagnosed as child abuse, orbital fracture, or other tumors such as rhabdomyosarcoma. CT and MRI can provide valuable diagnostic information for neuroblastoma identification, especially for pin pointing the primary site of the tumor. A giant hypoechoic mass with hyperechoic areas is the most common abdominal sonographic finding,^[16]^ which was observed in the present case. With the exception of case 11, abdominal CT and/or MRI scans were performed in all of the cases reviewed, and abdominal masses in the adrenal gland were often discovered. The radiographic findings in the orbits usually included thickened bones, periosteal reactions (speculated bones), and lytic defects. In the MRI scans, neuroblastomas usually present with heterogeneous low signal intensity on T1-weighted images and high signal intensity on T2-weighted images.^[6]^

The histopathological analyses of biopsied orbital masses were often performed to confirm the diagnosis of orbital neuroblastoma metastasis. The histological staining of the biopsied tissues revealed small round cells with a scanty cytoplasm and darkly stained nuclei.^[9,12]^ Moreover, neural markers, such as neuron-specific enolase, TH, and synaptophysin, were often immunopositive in the tumor tissues, while S100 and leukocyte common antigens were immunonegative (Table 1).^[12]^

The present patient refused any therapeutic regimen. At present, the primary treatment for neuroblastoma is chemotherapy. Multiple protocols with various agents, including carboplatin, vincristine, etoposide,^[8]^ cisplatin, doxorubicin, cyclophosphamide, and ifosfamide,^[12]^ are presently available. Other combined therapeutic strategies include 13-cis-retinoic acid,^[9]^ local radiation, resection of the periorbital tumor, and autologous hematopoietic stem cell transplantation. However, in case of more aggressive tumors, drug resistance should be resolved in the future. Targeting techniques by infusion of meta-iodobenzylguanidine conjugated to ^131^I and ^125^I are also promising for future developments.^[17]^

The prognosis for children with neuroblastoma is dependent on many factors, such as age at diagnosis, disease stage, and histological grade. Orbital neuroblastoma metastases are commonly associated with poor prognosis.^[4]^ Among the 11 cases with orbital neuroblastoma metastases, systemic chemotherapy was initiated in 6 cases. Although the symptoms improved by follow up after 4 months to 2 years in 4 patients, 2 children died after chemotherapy. Furthermore, although the survival chances for children diagnosed with neuroblastoma have markedly improved over the last 30 years with the advance of multiple treatment modalities, such as chemotherapy, surgery, stem cell transplantation, and immunotherapy,^[2]^ the survival rate for patients with neuroblastoma with orbital metastasis remains low.

## Author contributions

**Conceptualization:** Wan-Ju Yang, Yuan-Yuan Zhou, Shuang Li, Yi Xiang.

**Data curation:** Wan-Ju Yang, Yuan-Yuan Zhou, Fang Zhao, Zhong-Ming Mei, Shuang Li, Yi Xiang.

**Investigation:** Wan-Ju Yang, Yuan-Yuan Zhou, Fang Zhao, Zhong-Ming Mei, Shuang Li, Yi Xiang.

**Project administration:** Wan-Ju Yang, Yuan-Yuan Zhou, Shuang Li, Yi Xiang.

**Resources:** Wan-Ju Yang, Yuan-Yuan Zhou, Fang Zhao, Zhong-Ming Mei, Shuang Li, Yi Xiang.

**Supervision:** Wan-Ju Yang, Yuan-Yuan Zhou, Shuang Li, Yi Xiang.

**Validation:** Wan-Ju Yang, Yuan-Yuan Zhou, Fang Zhao, Zhong-Ming Mei, Shuang Li, Yi Xiang.

**Visualization:** Wan-Ju Yang, Yuan-Yuan Zhou, Fang Zhao, Zhong-Ming Mei, Shuang Li, Yi Xiang.

**Writing – original draft:** Wan-Ju Yang, Yuan-Yuan Zhou, Fang Zhao.

**Writing – review & editing:** Wan-Ju Yang, Yuan-Yuan Zhou, Fang Zhao, Zhong-Ming Mei, Shuang Li, Yi Xiang.

## References

[1] Bernstein ML Leclerc JM Bunin G A population-based study of neuroblastoma incidence, survival, and mortality in North America. J Clin Oncol 1992;10:323–9. 1732433 10.1200/JCO.1992.10.2.323

[2] Gutierrez JC Fischer AC Sola JE Markedly improving survival of neuroblastoma: a 30-year analysis of 1,646 patients. Pediatr Surg Int 2007;23:637–46. 17476512 10.1007/s00383-007-1933-7

[3] Kim S Chung DH Pediatric solid malignancies: neuroblastoma and Wilms’ tumor. Surg Clin North Am 2006;86:469–87. xi. 16580935 10.1016/j.suc.2005.12.008

[4] Smith SJ Diehl NN Smith BD Incidence, ocular manifestations, and survival in children with neuroblastoma: a population-based study. Am J Ophthalmol 2010;149:677.e2–82.e2. 20149339 10.1016/j.ajo.2009.11.027 PMC3905802

[5] Bay A Faik Oner A Raccoon eyes. Indian Pediatr 2005;42:949. 16208058

[6] Rao AA Naheedy JH Chen JY A clinical update and radiologic review of pediatric orbital and ocular tumors. J Oncol 2013;2013:975908. 23577029 10.1155/2013/975908 PMC3610355

[7] Kalaskar RR Kalaskar AR Neuroblastoma in early childhood: a rare case report and review of literature. Contemp Clin Dent 2016;7:401–4. 27630510 10.4103/0976-237X.188579 PMC5004559

[8] Cliff JF Newman L Malone M Facial features of widespread neuroblastoma: a case report. Int J Paediatr Dent 2001;11:215–20. 11484472 10.1046/j.1365-263x.2001.00269.x

[9] Varma D George N Livingston J Acute visual loss as an early manifestation of metastatic neuroblastoma. Eye (Lond) 2003;17:250–2. 12640418 10.1038/sj.eye.6700289

[10] Gumus K A child with raccoon eyes masquerading as trauma. Int Ophthalmol 2007;27:379–81. 17534581 10.1007/s10792-007-9089-y

[11] Moran DE Donoghue V Periorbital ecchymosis (’raccoon eyes’) as the presenting feature of neuroblastoma. Pediatr Radiol 2010;40:1710. 19946772 10.1007/s00247-009-1467-3

[12] Salmi D Patel C Imashuku S Neuroblastoma of unknown primary site with periorbital bone metastasis in a child. Pediatr Blood Cancer 2010;55:361–3. 20582979 10.1002/pbc.22524

[13] Muniz AE Metastatic neuroblastoma: the mimicker of basilar skull fracture in children. J Emerg Med 2012;42:e19–21. 19828277 10.1016/j.jemermed.2009.08.014

[14] Ghosh SK Dutta A Basu M Raccoon eyes in a case of metastatic neuroblastoma. Indian J Dermatol Venereol Leprol 2012;78:740–1. 23075644 10.4103/0378-6323.102370

[15] Kurian J Chen NN Shinder R Orbital metastatic neuroblastoma. Ophthalmology 2016;123:1674. 27450817 10.1016/j.ophtha.2016.03.044

[16] White SJ Stuck KJ Blane CE Sonography of neuroblastoma. AJR Am J Roentgenol 1983;141:465–8. 6603752 10.2214/ajr.141.3.465

[17] Haase GM Perez C Atkinson JB Current aspects of biology, risk assessment, and treatment of neuroblastoma. Semin Surg Oncol 1999;16:91–104. 9988866 10.1002/(sici)1098-2388(199903)16:2<91::aid-ssu3>3.0.co;2-1

